# Magnetizing Polymer Particles with a Solvent-Free Single Stage Process Using Superparamagnetic Iron Oxide Nanoparticles (SPION)s

**DOI:** 10.3390/polym14194178

**Published:** 2022-10-05

**Authors:** Björn Düsenberg, Philipp Groppe, Stephan Müssig, Jochen Schmidt, Andreas Bück

**Affiliations:** 1 Institute of Particle Technology, Friedrich-Alexander-Universität Erlangen-Nürnberg, Cauerstraße 4, D-91058 Erlangen, Germany; 2 Collaborative Research Center 814—Additive Manufacturing (DFG, German Research Foundation), Am Weichselgarten 9, D-91058 Erlangen, Germany; 3 Department of Chemistry and Pharmacy, Inorganic Chemistry, Friedrich-Alexander-Universität Erlangen-Nürnberg, Egerlandstraße 1, D-91058 Erlangen, Germany

**Keywords:** superparamagnetic iron oxide nanoparticles (SPION), polymer, dry coating, solvent-free process, discrete core-shell coating

## Abstract

Magnetic polymer composites are used in a variety of applications in many industries. Their production methods are usually time-consuming and solvent-intensive as they are performed in liquid phase processes, such as emulsion polymerization or precipitation. In this work, a quick, easy, and solvent-free method is presented to coat polymer particles with a discrete, non-coherent coating of superparamagnetic nanoparticles. The results of the dry coating process are evaluated optically, by means of scanning electron microscopy (SEM), via powder X-ray diffraction and thermally by means of differential scanning calorimetry, before finally demonstrating the effectiveness of dry coating by means of a vibrating sample magnetometer.

## 1. Introduction

Magnetic polymer composites are being investigated and used for a wide variety of applications in the medical technology, for example magnetic resonance spectroscopy [1,2], pharmaceutical [3,4,5,6,7], and (bio-)chemical industries [8,9,10] as gas and humidity sensors [11,12] or for urea sensing [13]. The sensing abilities of iron oxides are always of interest, as recently iron oxide—based supra-particles were used to track the temperature [14] or to create a magnetic fingerprint [15]. Therefore, the functionalization and production of magnetic polymers has been the topic of research for several years. A number of processes have been developed, all taking place in the liquid phase, where the polymerization occurs in a liquid or emulsion containing the magnetic particles [16,17,18,19,20,21] as well as the deposition of nanoscale iron oxid within the liquid phase [22]. This not only entails the use of various solvents, which can be problematic for both humans and the environment, but this process route is always followed by a drying step, which also takes time and resources.

To create a magnetizable polymer composite, various substances are currently used; most of them are iron-based, since iron has the highest saturation magnetization at ambient temperatures [23]. Iron oxides, especially Fe_3_O_4_ [21,24,25,26,27,28,29,30,31,32] and γ-γ-Fe_2_O_3_, are the most popular. The benefits of iron oxides in comparison with other iron compounds are their stability, their environmental friendliness, and their inexpensiveness [33], which makes them easy-to-use and to prepare [34,35,36,37,38,39]. Their combination with polymers can yield completely new functionalities such as the recently reported magnetic indication of elapsed temperature events [33]. Another large group of magnetic particles with which the polymer is modified during polymerization are the carbonyl iron compounds such as shown in [40,41,42,43,44,45,46,47,48,49]. As well as some rarer compounds containing zinc [50], copper [51], or cobalt [52].

Compared to the wet chemical methods mentioned, dry coating (DC) offers several advantages, especially the absence of solvents, possible stabilizers within the liquid phase, and therefore the non-required drying step, making dry coating a sustainable and cost-efficient method of functionalization. Dry coating has been used for some time to improve the flowability [53,54] and fluidizability [55] of powders, e.g., by uniform deposition of silica nanoparticles on the surface of the particles. Research has also been conducted to use DC for the production of customized advanced materials, for example to control the powder charge [56,57], improve the humidity resistance [58] or to increase the electrical conductivity [59]. The influencing factors for dry coating can be found on the process side as well as on the material side. On the process side, the following variables should be mentioned: the filling level of the mixer, the ratio of HP to GP to mixing aids, the process temperature [60], and the mechanical energy input (stirrer speed). On the material side, the particle sizes of the materials used, as well as the differentiation between conductive and non-conductive materials and the densities have an influence on the process result [61].

**Figure 1:**
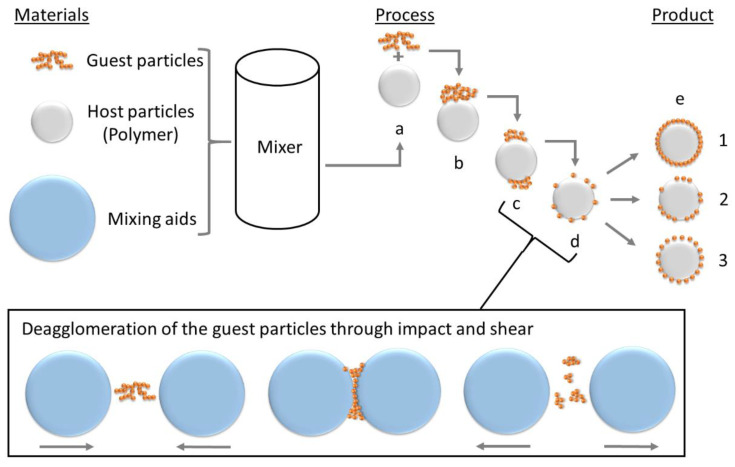
Process of dry coating, described by Alonso et al. [62,63,64]. (**a**) addition of GP to HP and first contact (**b**); (**c**) deagglomeration and (**d**) dispersion of the GP; (**e**) different product qualities to achieve.

The process, inherently designed to deposit nanoparticles (guest particles—GP) on host particles (HP), is based on the adhesion between those resulting from van-der-Waals forces and consists of several steps that transition into each other as can be seen in Figure 1. In step (a) host and guest particles are added to the mixing device and brought into contact. (b) Guest particles are dispersed (c) and deagglomerated (d) by assistance of mixing aids. Due to the impact and shear between the mixing aids, the nanoparticle agglomerates are exposed to constant mechanical energy. The agglomerates are broken down by the mechanical energy so that increasingly smaller aggregates can attach to the host particles. Ideally, single nanoparticles are obtained during deagglomeration. (d) The different results which can be obtained by dry coating are schematically described in (e): ideal hexagonal coating is a purely theoretical ideal condition (1). The random coating occurs during a non-optimized process, where the product already has the desired properties (2). The ideal random coating occurs in an optimized process, where the smallest possible amount of additive achieves the desired product properties due to a close-to-ideal coating quality. It is important to notice, that powder dry coating can be performed in several kinds of mixing devices and is not limited to a shaker mixer, as used in this work and therefore even big batches can be produced.

For an ideal random single-layer coating, as in Figure 1 (e-1), Yang et al. [53] gave the approximate additive content in weight percent:

(1) $$m_{g,wt\%}=\frac{\left(N\times d_{g}^{3}\times \rho_{g}\right)}{\left(d_{host}^{3}\times \rho_{host}\right)+\left(N\times d_{g}^{3}\times \rho_{g}\right)}\;\cdot \;100$$

with the diameter of the host particles *d_host_* and the guest particle diameter *d_g_* as well as the density of the host particles *ρ_host_* and the guest particles *ρ_g_*, as well as *N* (cf. Equation (2)), which represents the number of guest particles to achieve the surface coverage.

(2) $$N=\frac{4{\left(d_{host}+d_{g}\right)}^{2}}{d_{g}^{2}}$$

**Figure 2:**
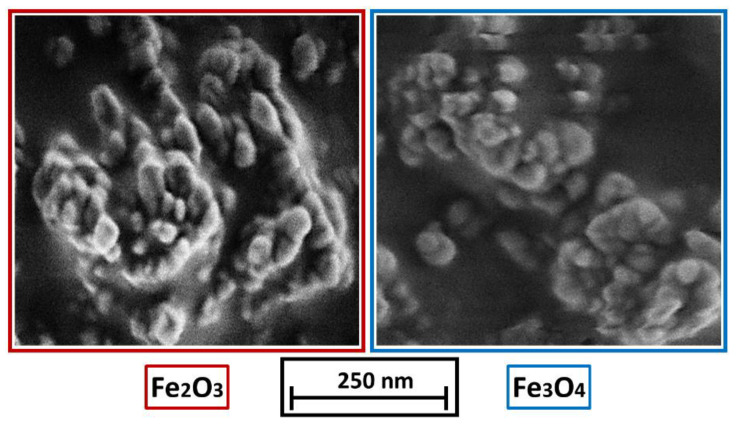
(**left**): γ-Fe_2_O_3_ nanoparticles, (**right**): Fe_3_O_4_ nanoparticles.

**Figure 3:**
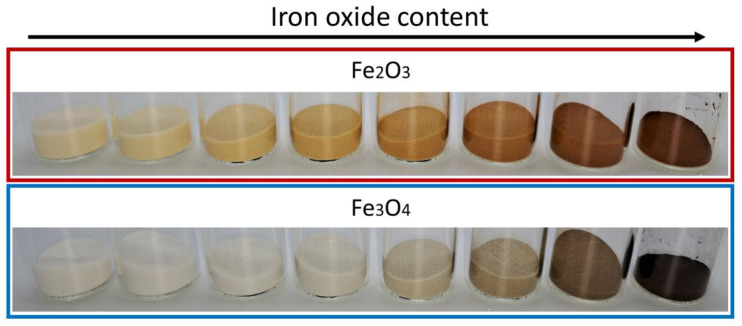
Change in color of the coated powders. From left to right, increase of iron oxide content from 0.05 to 5 wt.% according to experiments listed in Table 1. The last beaker in each row (most right position) shows the pure SPIONs.

The dry coating process will therefore form a discrete core—shell coating around on the particle surface. When the process is optimized a uniformly distributed coating, which is near Figure 1 (e-3), is achievable. The difficulties in dry coating are primarily the de-agglomeration of the nanoparticulate GP, selecting the process time so that de-coating does not occur, and observing the yield, since triboelectric effects have a massive influence on this.

The aim of this work is therefore to showcase a sustainable, solvent-free, scalable, and easy-to-use method to create discrete magnetic core-shell structures on polypropylene (PP) particle surfaces by use of dry particle coating. PP is a widely used thermoplast with high chemical resistance and good thermal properties which is used in additive manufacturing or injection moulding. A magnetisable PP-composite would, for example, be perfect to produce magnetically responsive sensors. The composite is analyzed by use of scanning electron microscopy to showcase the coating and the differences in the two additives used, as well as to evaluate the degree of coverage on the polymer surface. Furthermore, the powder is analyzed by powder X-ray diffraction to evaluate polymorphs in the coating material and differential scanning calorimetry to determine the crystallinity of the polymer- super paramagnetic iron oxide nanoparticle (SPION) composite. Finally, the magnetizability of the newly made composites is determined and results are compared to each other.

## 2. Materials and Methods

Polypropylene, type Coathylene PD0580 (Axalta, Bulle, Switzerland) with a density of 0.907 g cm^−3^ and particle sizes of x_10.3_ = 32 ± 3 µm, x_50.3_ = 93 ± 5 µm and x_90.3_ = 182 ± 4 µm (measured by laser diffraction (Mastersizer 2000, Malvern, UK)) is used. Magnetic γ-Fe_2_O_3_ powder, 98%, (NanoArc^®^, APS Powder, S.A, Singapore) with a particle diameter between 20–40 nm and a specific surface area (BET) of 32.188 m^2^ g^−1^ is used as an additive and compared with self-synthesized Fe_3_O_4_, with a mean particle size between 10 to 20 nm and a specific surface area (BET) of 79.138 m^2^ g^−1^, stabilized with oleic acid according to [14]. The synthesis of Fe_3_O_4_ is achieved by a co-precipitation reaction. Therefore, FeCl_3_ 6 H_2_O (total: 10.80 g, 40 mmol, Sigma Aldrich, Darmstadt, Germany, >99%) and FeCl_2_ 4 H_2_O (total: 3.98 g, 20 mmol, Fluka—obtained by Sigma Aldrich, Darmstadt, Germany, >99%) were dissolved in deionized water (225 mL) at room temperature (ca. 20 °C) and mixed with 30% aqueous ammonia solution NH_3_ (aq.) (25 mL). After 60 s of stirring, the black precipitate was magnetically separated, and the overlaying water decanted. In this manner, the particles were washed with deionized water (250 mL) three times before dispersing them in water (250 mL). The self-synthesized nanoparticle dispersion is dried with a vacuum oven and pre-crushed before use via mortar and pestle. The commercial γ-Fe_2_O_3_ nanoparticles as well as the self-synthesized Fe_3_O_4_ nanoparticles are displayed in Figure 2. It is important to note, that the Fe_3_O_4_ powder is difficult to deagglomerate and most Fe_3_O_4_ particles are present in agglomerates larger than 100 nm.

### 2.1. Dry Coating

A Turbula mixer (T2f, Willy A. Bachofen AG, Muttenz, Switzerland) is used for the coating experiments. The polymer coating takes place in borosilicate snap-on lid glasses with a volume of 25 mL. To ensure reproducibility, each experiment is performed 3 times. The mixer is operated in all experiments at 49 rpm with a coating time of 60 min.

In each experiment 3 g of polymer powder were used. To achieve deagglomeration of the guest particles, 9 g of glass spheres (Sili S, Sigmund Lindner GmbH, Warmensteinach, Germany) with particle size ranging between 1.0–1.25 mm and a bulk density of 1.5 g cm^−1^ were added as mixing aids. The glass beads were separated with a 0.8 mm sieve after the coating process.

### 2.2. Nitrogen Sorption

Nitrogen sorption measurements (77.4 K) were performed to determine the properties of the nanoparticles using a NovaTouch LXII (Anton Paar, Graz, Austria). Before each measurement, the samples were gassed out at 200 °C for 12 h at vacuum and weighted in an inert atmosphere.

### 2.3. Scanning Electron Microscopy

The polymer particles have been characterized by scanning electron microscopy (SEM) using a Gemini Ultra 55 (Zeiss, Jena, Germany) device equipped with a SE2 detector. An acceleration voltage of 1 kV has been applied.

### 2.4. X-ray Diffraction

For structural analysis of the product, powder X-ray diffraction (XRD) was performed with an AXS D8 Advance diffractometer in the Bragg–Brentano geometry (Bruker, Billerica, MA, USA). The device is equipped with a VANTEC-1 detector and a Ni filter and uses Cu Kα radiation (154 pm). The step size for collecting the diffractograms was set to 0.014° with a measuring time of 1 s per step for the range of 2Θ = 10–60°.

### 2.5. Dynamic Scanning Calorimetry

The crystallinity and the crystallization temperature of the coated powders are determined by differential scanning calorimetry (DSC). For this purpose, a Polyma 214 (Netzsch, Selb, Germany) is used. The samples with a weight of 10 mg ± 0.1 mg are measured with covered aluminum pans type Concavus Lids (Al), NGB817526 (Netzsch, Germany) with dry nitrogen gas purging at 40 mL min^−1^. As the melting temperature of the PP is at 186 °C, the temperature profile to measure the thermogram is as following: (1) Start at 20 °C, (2) heating to 200 °C by a gradient of 10 K min^−1^, (3) isothermal step of 60° s, (4) cooling by a gradient of 10 K min^−1^.

### 2.6. Virbating Sample Magnetometer

Magnetic properties of the nanoparticles as well as the product particles were determined with a vibrating sample magnetometer (VSM) type VersaLabTM 3T from Quantum Design Inc., San Diego, CA, USA. Field dependent magnetization measurements were conducted between −30 to +30 kOe with a measurement speed of 50 Oe s^−1^ and 5 Oe s^−1^ between −5 and 5 kOe.

## 3. Results

### 3.1. Degree of Coverage

The effect of dry coating can be observed visually in Figure 3. The surface coating noticeably changes the color of the powder to the color of the iron oxide used. Inside the red box (top row), the powders functionalized with Fe_2_O_3_ are displayed; in the blue box (bottom row) results of functionalization with Fe_3_O_4_ are displayed.

**Figure 4:**
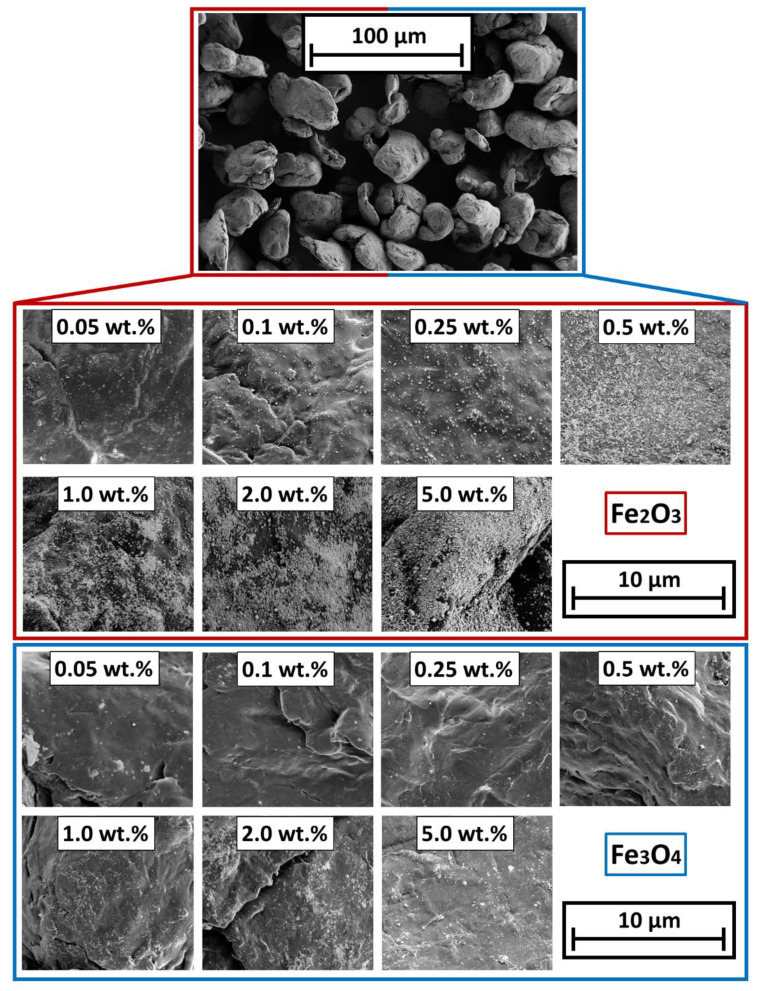
SEM micrographs of the different SPION coated polymer surfaces. (**Top**): Overview of the particle bulk; (**Bottom**): coated surfaces.

The evaluation of the particle surface by means of SEM shows, an increase in the degree of coverage with an increase of the SPION content. Figure 4 shows the change in the degree of coverage of Fe_2_O_3_ (red box) and Fe_3_O_4_ (blue box). The coating with Fe_3_O_4_ is not as uniform as in the samples with γ-Fe_2_O_3_. The reason is found in the production process of the SPIONs by spray drying: The Fe_3_O_4_ formulation has not been stabilized against aggregation of the primary particles while drying. Nevertheless, the images show a homogeneous, discrete distribution of the guest particles on the polymer surface. Due to the van der Waals forces acting between the GP and the HP, the two materials are almost inseparably bonded, which makes the process stand out. Due to the surface deposition of the GP on the HP, their effect, such as magnetization in this case, is not hindered by any polymer layers, as often occurs in liquid phase processes, where the GP preferentially accumulate inside a polymer matrix. In that case, the polymer would act as a spacer between the nano particles and the external magnetic field.

**Figure 5:**
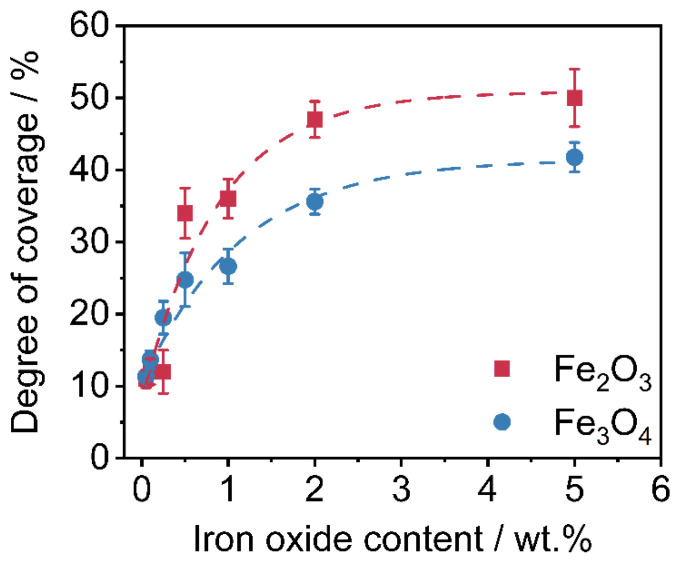
Degrees of coverage of the nanoparticles on the polymer surface (*n* = 3).

Corresponding to the SEM-images from Figure 5, the degrees of coverage (see Figure 4) are determined. Image analysis via MATLAB, due to binarization of the SEM images by use of Otsu’s method [65] to determine the threshold. After binarization, the guest particles are displayed in white and the polymer surface is displayed in black. The degree of coverage is the amount of white area within the image. It is easy to observe that the degree of coverage increases continuously and flattens around 2 wt.% additive content onwards. The lower degree of coverage of the Fe_3_O_4_ samples is also evident here. The higher degree of coverage when using the γ-Fe_2_O_3_ is related to the easier deagglomeration of the commercial product. The Fe_3_O_4_ particles from our own production, were hydrophobized by the oleic acid but not functionalized against aggregation. Results indicate that the interparticulate forces between the Fe_3_O_4_ primary particles are stronger than the forces needed to deagglomerate them sufficiently during the coating process, leading to a non-ideal coating result and even spread on the HP surface.

**Figure 6:**
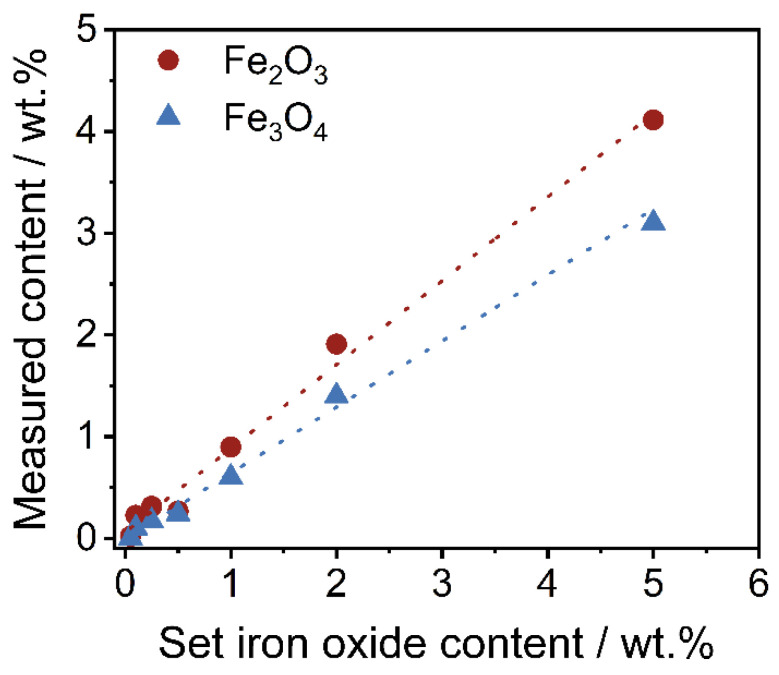
Measured iron oxide content in regard to the set iron oxide content of both formulations.

To confirm the lower degree of coverage of Fe_3_O_4_, a thermo-gravimetrical experiment to determine the amount of SPIONs on the polymer powder has been carried out. For this purpose, around 1 g of polymer-iron oxide powder is weighed into a ceramic bowl. Afterwards, the polymer is burned at 900 °C for 10 min within an oven. Subsequently, the residual mass (the inorganic iron oxide) is weighed, and the measured iron oxide content is calculated. The results are shown in Figure 6. As displayed, the residual mass of Fe_3_O_4_ within the polymer-iron oxide composite is lower in comparison with the easily-dispersible commercial product.

It is not surprising that the measured iron oxide content is lower than the set content in this work, as losses always occur on the wall of the mixing device. For the purpose of this work and better comparability, further results are displayed in regard to the set iron oxide content.

### 3.2. X-ray Diffraction

The measurements are intensity min-max-normalized for all values obtained within a sample according to:

(3) $$x^{\prime }=\frac{x-\min\left(x\right)}{\max\left(x\right)-\min\left(x\right)}$$

**Figure 7:**
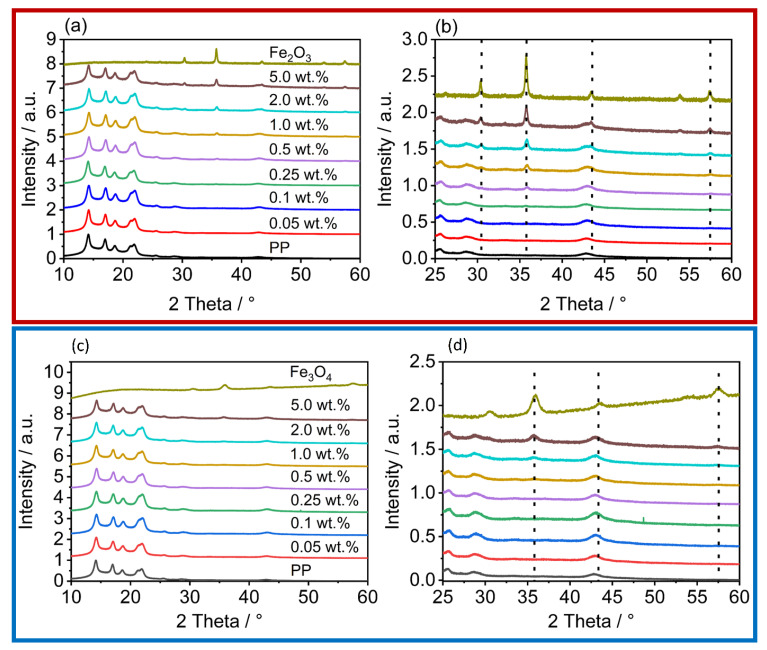
XRD of the iron oxides, the unfunctionalized PP, and the coated powders; the red box contains the diffractograms of the γ-Fe_2_O_3_ coated powders, the blue box the diffractograms of the powders with Fe_3_O_4_. (**a**,**c**) shows an overview over the measured area; (**b**,**d**) zooms inside the area, where the increase of the iron oxide reflexes are observed.

In all diffractogramms (Figure 7), the increase in the reflexes, characteristic of the iron oxides used, can be clearly observed. As can be seen in the diffractograms from the reflexes 2Θ = 31°, 2Θ = 38°, 2Θ = 47°, and 2Θ = 55°, the iron oxide nanoparticles are not one pure polymorph, but exhibit both alpha (hematite) and gamma (maghemite) reflexes [66,67]. The reflexes correspond to those of the raw material used, which proves the gentle processing conditions during dry coating, as otherwise a change in the polymorphy [66] would have occurred. Although not as distinct, the same behavior can be seen in the polymer particles coated with Fe_3_O_4_. The polymorphy is detectable on the surface. The reason for this is that there is often a slight transformation on the nanoparticle surface where γ-Fe_3_O_4_ converts to Fe_2_O_3_, but the Fe_3_O_4_ is still present in the core [68].

### 3.3. Thermal Analysis

**Figure 8:**
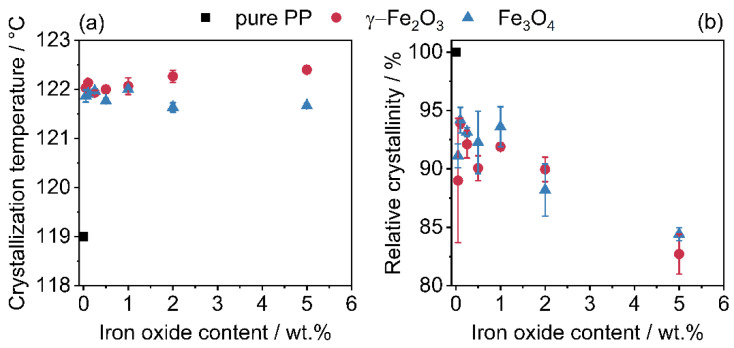
Change in crystallization temperature (**a**) and relative crystallinity (**b**) due to additivation with iron oxide nanoparticles. The relative crystallinity refers solely to the weight of the polymer, the SPIONs have been excluded.

Thermal analysis by DSC shows the influence of the iron oxide nanoparticles on the crystallization temperature (Figure 8a) as well as the relative crystallinity (Figure 8b). Further processing, e.g., in additive manufacturing or injection moulding, strongly depends on the melting and crystallization behavior. Figure 8 shows the determined crystallization temperatures (Figure 8a) and the corresponding relative crystallinities (Figure 8b). The nanoparticles act as crystallization nuclei and, thus, shift the crystallization temperature as expected to higher temperatures [69,70,71]. This effect is further enhanced by the higher heat transfer ability of the iron oxides. The more rapid heat transfer also explains the reduced relative crystallinity, since colder temperatures are also passed.

### 3.4. Vibrating Sample Magnetometer

**Figure 9:**
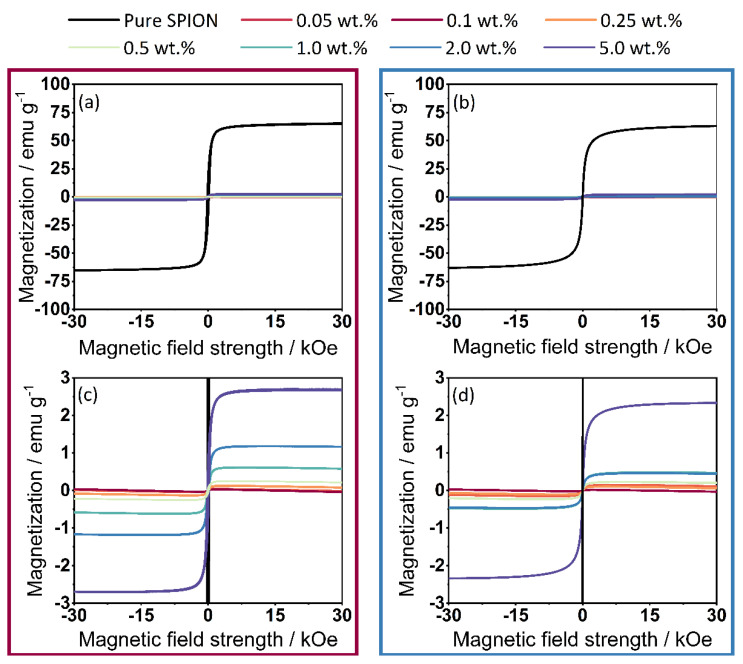
Measurement results from VSM. (**a**) Results from the complete measurement spectrum of γ-Fe_2_O_3_, (**b**) Results from the complete measurement spectrum of Fe_3_O_4_, (**c**) Magnification; (**d**) Magnification of the magnetization—(values displayed in cgs units).

The results of the magnetometer measurements (Figure 9) show the full spectrum of magnetization measurements of the γ-Fe_2_O_3_ (a-1) and Fe_3_O_4_ (b-1) samples, showing that the maximum magnetization of the commercial Fe_2_O_3_ SPIONs does not differ from that of the synthesized Fe_3_O_4_ SPIONs. In the enlarged plot of the data (a-2) for γ-Fe_2_O_3_ and (b-2) for Fe_3_O_4_ functionalization, it is shown that the magnetization by the commercially available SPIONs is higher than with the self-synthesized Fe_3_O_4_. In this case, this is in line with expectations, since it has already been shown in the determination of the degree of coverage (Figure 4) that the coating process using Fe_3_O_4_ cannot be performed optimally. A stabilization of the SPIONs that keeps the Fe_3_O_4_ nanoparticles separated during the drying step will solve this problem. Since the functionalization, unlike in liquid-phase processes, only takes place on the surface of the polymer, the resulting value of the magnetization is comparatively low. During dry coating, much less iron oxide is required to functionalize the polymer. For example, in [26], in which the ration between polymer and SPIONs is around 1:3, or in [28] where SPION contents up to 80% are used. For the iron oxides used, the more additive is applied to the surface, the higher the magnetization.

**Figure 10:**
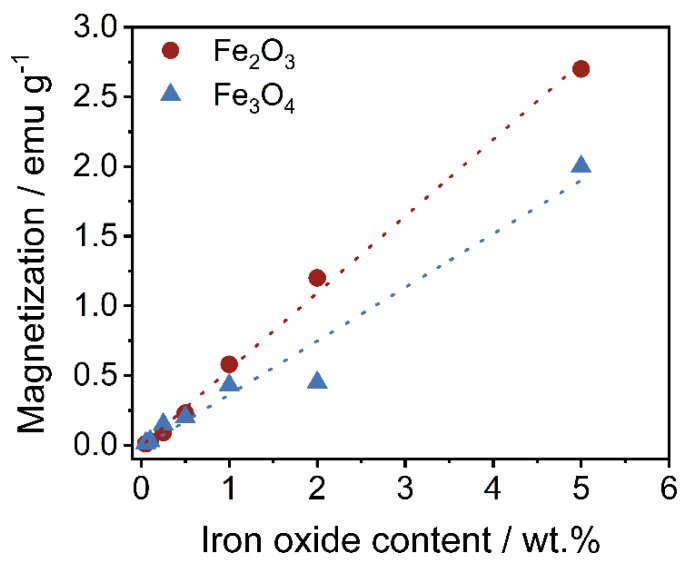
Relationship between the maximum magnetization as regards the set iron oxide content.

If the maximum magnetizations are extracted from the measurements (Figure 9) and plotted against the set iron oxide content (Figure 10), an expected linear relationship can be seen as the magnetization scales with the amount of iron oxide available. Again, the maximum values of Fe_3_O_4_ are slightly lower than those of Fe_2_O_3_, which is again due to the different coating quality.

## 4. Conclusions

In this work, a solvent-free dry coating process for producing magnetic polymer particles is introduced. Seven different additive concentrations of γ-Fe_2_O_3_ and Fe_3_O_4_ were used to show the versatility of this process as it is possible to coat commercially available host particles quickly and sufficiently. The discrete coating is evenly distributed on the polymer particle surface and adjustable by the additive concentration. The additive content controls the magnetization directly. Dry coating is therefore a simple, sustainable coating method for the production of magnetizable polymer composites with commercially available products and a low need for resources.

## Figures and Tables

**Table 1:** Plan of coating experiments.

No.	γ-Fe_2_O_3_ wt.%	Fe_3_O_4_ wt.%
1	0.05	-
2	0.1	-
3	0.25	-
4	0.5	-
5	1.0	-
6	2.0	-
7	5.0	-
8	-	0.05
9	-	0.1
10	-	0.25
11	-	0.5
12	-	1.0
13	-	2.0
14	-	5.0

## Data Availability

Data is available upon reasonable request.
